# MEK + CDK4 a regimen for non-BRAF V6000 melanoma

**DOI:** 10.1186/1479-5876-13-S1-K9

**Published:** 2015-01-15

**Authors:** Jeff Sosman

**Affiliations:** 1 Vanderbilt-Ingram Cancer Center, Nashville, Tennessee, USA

## 

Targeting of the BRAF^V600^ mutation has proven a highly effective treatment strategy for the subset of melanoma carrying this mutation. However, the majority (~60%) of patients with advanced melanoma do not carry this mutation and are not targetable by selective BRAF inhibitors. The rapidly evolving genetic landscape of melanoma has revealed that the vast majority of *non-V600 BRAF* mutant melanoma still express other genomic alterations that lead to MAP kinase pathway activation. The most frequent of these are activating mutations in the *NRAS* gene representing 1/3 of the *non-V600 BRAF* mutant melanoma or 15-20% overall. The other MAPkinase activating genetic alteration include *NF1* loss of function, *MAP2K1*, *C-RAF*, *K-* or *H-RAS*, and alternate -non-V600 mutations in the *BRAF* gene. Therefore MEK inhibitors, ERK inhibitors, or even non-selective RAF inhibitors may represent effective therapeutic interventions. Early results in the NRAS mutant and non-*NRAS* or *BRAF V600E* mutant melanoma demonstrate clinical activity but likely not as robust as hoped. A trial with a MEK inhibitor (binmetinib) has demonstrated an unconfirmed objective response rate of approximately 20%. MEK inhibitors are also active against non-*NRAS*, *non-BRAF V600* mutant melanoma including responses in those melanomas carrying mutations in BRAF gene outside of V600 codon.

Genomic findings also demonstrate a high frequency of dysregulation of cell cycle regulatory proteins including genomic alterations in *CDKN2A*, *CDK4*, *Cyclin D1*, etc.

This finding suggests a role for CDK4/6 inhibitors in the therapy of melanoma. Additionally, preclinical work has demonstrated the effectiveness of the combination of MEK inhibitors (trametinib, binimetinib) with CDK4/6 inhibitors (palbociclib, LEE011) in multiple mouse models of *NRAS* mutant melanoma. New, selective, and more effective CDK4/6 inhibitors have arrived in the clinic including palbociclib and LEE011 to name several, Based on this background clinical investigations have recently begun in non-BRAF V600 mutant melanomas with combination MEK inhibitor and CDK4/6 inhibitor treatment. While very early in their development, Binimetinib and LEE011 has already produced objective clinical responses in about 1/3 of NRAS mutant melanoma patients with another 1/3 demonstrating clinical responses not meeting RECIST 1.1 criteria. Most impressive is the rapid nature of these responses and in some cases accompanying relief from tumor-related symptoms. Early on some significant toxicities have been observed and alternate schedules are being explored. These regimens are also being evaluated in the non-NRAS mutant, non BRAF V600 mutant melanomas. As premature as these results are they still provide excitement that targeting mutations other than BRAF^V600^ is feasible and may provide hope for additional therapeutic options for the majority of melanoma patients.

